# Feasibility of a Systematic Same-Day Discharge Approach After Elective Mitral Transcatheter Edge-to-Edge Repair

**DOI:** 10.1016/j.shj.2024.100395

**Published:** 2025-01-18

**Authors:** Guillaume Leurent, Alexandra Audren, Guillaume L’Official, Grégoire Le Gac, Nicolas Nesseler, Hervé Corbineau, Erwan Donal, Vincent Auffret

**Affiliations:** a Department of Cardiology, Univ Rennes 1, CHU Rennes, Inserm, LTSI - UMR 1099, Rennes, France; b Department of Anesthesia and Critical Care, Pontchaillou, University Hospital of Rennes, Rennes, France; c CHU Rennes, Division of Thoracic and Cardiovascular Surgery, Rennes, France

**Keywords:** M-TEER, Same-day discharge, Safety

## Abstract

• Same-day discharge after elective mitral-transcatheter edge-to-edge repair (M-TEER) can be proposed in a selected patient population.
• Same-day discharge after elective M-TEER seems safe in a selected patient population.
• Around one patient out of 5 may be eligible for a same-day discharge management after M-TEER.

Mitral transcatheter edge-to-edge repair (M-TEER) is usually performed in the setting of a hospitalization. Some recent data suggest the safety of a same-day discharge (SDD) approach after M-TEER, but its applicability remains unknown, particularly for patients under general anesthesia.^1^, ^2^, ^3^ Thus, we thought to prospectively evaluate the feasibility of a systematic SDD approach for patients who are candidates for elective M-TEER at our center over 1 year (March 2023 to March 2024).

All patients referred to our high-volume center (University Hospital of Rennes; France) for a heart team-validated M-TEER procedure were considered for an SDD approach if they met all the following criteria, in this order: (i), elective M-TEER; (ii), absence of medical contraindication (e.g., poor left ventricular function, clinically decompensated congestive heart failure, severe extracardiac comorbidities, indication for combined transcatheter aortic valve repair); (iii) voluntary patient, with good social support and Katz frailty score = 6. In elderly patients, specialist geriatric advice was generally required.

Our optimized organization led us to admit early in the morning fasting patients eligible for SDD to the catheterization laboratory with the intention of being the first patient of the day. The M-TEER procedure was standard, under general anesthesia, with echo-guided femoral venous catheterization and use of one Proglide (Abbott Medical) for venous closure. Anesthesia was discontinued at the end of the procedure, with an extubation in the catheterization laboratory. No protamine was given at the conclusion of the procedure. Given the hemodynamic stability during and after the procedure and the absence of femoral access problems, the patient was allowed to stand up 6 hours after the end of the procedure. A transthoracic echocardiography was performed to assess the M-TEER quality result and excluded delayed complications. Discharged was then authorized after a medical assessment of the patients’ full recovery. Patients were systematically called the following day. Finally, for this analysis, the last follow-up was collected by consulting the medical file or calling the attending physician or cardiologist.

Eighty M-TEER procedures were performed at our center using the Mitraclip system (Abbott Medical) during the study period, encompassing 15 (18%) patients (8 males, mean age 77 ± 9, mean Society of Thoracic Surgeons score 3.0 ± 3) eligible for an SDD approach (Figure 1). One patient, with early single leaflet device attachment despite a good initial M-TEER result, was admitted for a 24-hour monitoring. Thus, 14 (17%) patients were discharged the same day after the M-TEER procedure. Of note, no device was implanted in another patient due to a complex subvalvular apparatus preventing a satisfactory correction of the mitral regurgitation (unsuitability not understood before the intervention). However, this patient was discharged the same day and referred for mitral surgery.

**Figure 1:**
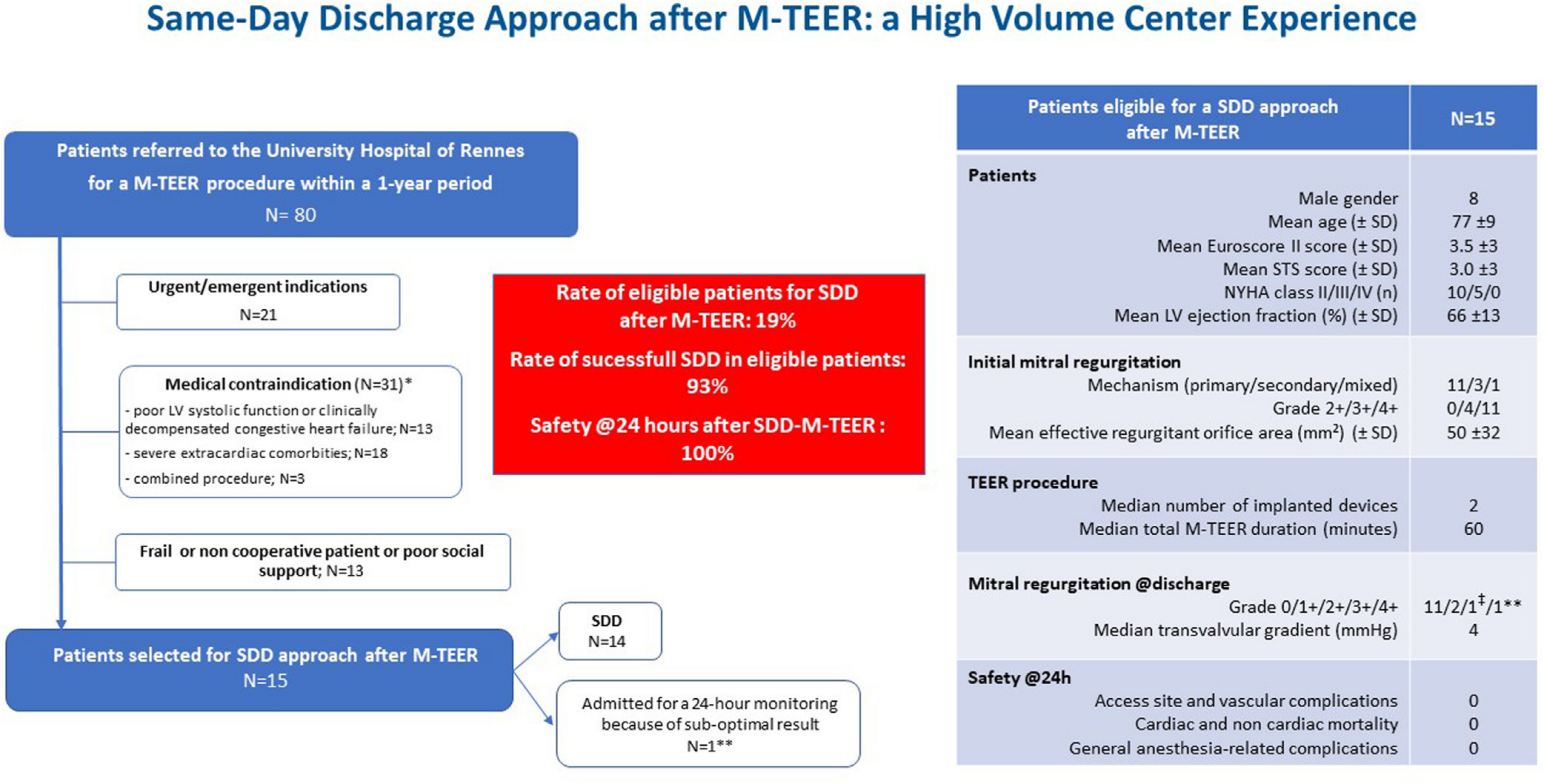
**Flowchart and key baseline characteristics of patients eligible for a same-day discharge after mitral transcatheter edge-to-edge repair.** ∗Patient can have several medical contraindications. ∗∗Transthoracic echo at discharge showed a recurrence of mitral regurgitation (grade 4) due to an early single leaflet device attachment. The patient was admitted for a 24-hour monitoring. ^‡^Technical failure: since a good result was not obtained, no device was implanted. The patient was, however, same-day discharged and referred for mitral surgery. Abbreviations: LV, left ventricle; M-TEER, mitral-transcatheter edge-to-edge repair; NYHA, New York Heart Association; SDD, same-day discharge; STS, Society of Thoracic Surgeons.

At 24 hours, none of the patients effectively discharged the same day had any access-site and vascular complications, cardiac and noncardiac mortality, or complications related to the general anesthesia. Median follow-up was 3 [2-14] months. None of the patients experience any delayed adverse events related to the procedure. The two patients with the procedure failure were hospitalized for heart failure. Among the 13 patients with a successful M-TEER procedure, New York Heart Association class was I and II in 10 and 3 patients, respectively.

To the best of our knowledge, this is the first report of a prospective and systematic evaluation of an SDD approach after M-TEER. Over 12 months, 18% of patients referred to our center for M-TEER were potentially eligible for SDD management. In patients actually discharged the same day after a successful M-TEER procedure, SDD appears feasible and safe.

Compared to a standard approach, SDD reduces bed occupancy, hospitalization costs, and the impact on patient oucomes.^4^ A dramatic increase in the volume of M-TEER procedures can be expected over the next few years. We are therefore convinced that such management should be encouraged.

However, eligible patients for SDD should be carefully selected in order to limit the risk of adverse events, such as afterload mismatch or delayed impairment after general anesthesia. Moreover, the postprocedural clinical and echocardiographic assessment must pay particular attention to the M-TEER result and to any complication, such as single leaflet device attachment, atrial shunting, or pericardial effusion.

Several limitations need to be recognized. This is a high-volume, single-center experience, including a highly selected, low-risk patient population. Further, larger-scale studies will be needed to confirm this approach as a standard of care. Because it was the only one reimbursed in France during the inclusion period, only the Mitraclip system was implanted. However, similar results can be expected with any other M-TEER device.

## Ethics Statement

The authors attest they comply with human studies committees and animal welfare regulations of the authors’ institutions and Food and Drug Administration guidelines, including patient consent when appropriate.

## Funding

The authors have no funding to report.

## Disclosure Statement

Guillaume Leurent reports proctoring activity and consultant fees from Abbott Medical and Edwards LifeSciences. Alexandra Audren reports speaker and consultant fees from Abbott Medical, Edwards LifeSciences, and Medtronic. Vincent Auffret reports speaker fees from Edwards LifeSciences and Medtronic and consultant fees from Medtronic and Boston Scientific. The other authors had no conflicts to declare.

## References

[1] Marmagkiolis K., Dogu Kilic I., Ismail A., Kose G., Iliescu C., Cilingiroglu M. (2021). Feasibility of same-day discharge approach after transcatheter mitral valve repair procedures. J Invasive Cardiol.

[2] Chowdhury M., Buttar R., Rai D. (2021). Same-day discharge after transcatheter mitral valve repair using MitraClip in a tertiary community hospital: a case series. Eur Heart J Case Rep.

[3] Tamburino C., Buccheri S., Popolo R.A. (2017). Feasibility and predictors of early discharge after percutaneous edge-to edge mitral valve repair. Heart.

[4] Krishnaswamy A., Isogai T., Brilakis E.S. (2023). Same-day discharge after elective percutaneous transcatheter cardiovascular interventions. JACC Cardiovasc Interv.

